# Special Issue “New Perspectives in Cardiovascular Surgery”

**DOI:** 10.3390/jcm11154535

**Published:** 2022-08-03

**Authors:** Annalisa Bernabei, Francesco Nicolini, Eduard Quintana, Alessandra Francica, Francesco Onorati

**Affiliations:** 1 Department of Cardiac Surgery, University Hospital of Verona, 37126 Verona, Italy; annalisa.bernabei@univr.it (A.B.); alessandrafrancica@yahoo.it (A.F.); 2 Cardiac Surgery Unit, Parma University Hospital, 43125 Parma, Italy; francesco.nicolini@unipr.it; 3 Department of Cardiovascular Surgery, Hospital Clinic de Barcelona, University of Barcelona, 08036 Barcelona, Spain; equintan@clinic.cat

In recent decades, cardiovascular surgery has been making great strides in the field of medicine. This editorial aims to give an overview of a selection of the several scientific papers that contributed to the Special Issue of the *Journal of Clinical Medicine*, “New Perspectives in Cardiovascular Surgery”.

This Special Issue provides new insights into the outcomes of widely performed surgical techniques such as septal myectomy. Pruna-Guillen [1], in his paper “Outcomes of Septal Myectomy beyond 65 Years, with and without Concomitant Procedures”, questioned the results of this procedure in patients older than 65 years old. Surprisingly, the survival at 2 years was 98%, confirming the safety of septal myectomy for hypertrophic cardiomyopathy even in fragile elderly patients. Although septal myectomy remains paramount to managing left ventricular flow (LVOT) obstruction, Affronti and colleagues [2] accurately reviewed the entire spectrum of possible surgical techniques beyond septal myectomy to explain their pathophysiologic rationale in the case of LVOT due to abnormalities in the systolic anterior motion or mitral valve/subvalvular apparatus.

The Ozaki procedure has been deeply analyzed by Gardin et al. [3]. This innovative technique consists of reconstructing the aortic valve with autologous pericardium. The team aimed to perform, for the first time, a morphological analysis of the glutaraldehyde-fixed pericardial tissue used in the Ozaki technique, demonstrating endothelial cell repopulation and, therefore, its non-cytotoxicity nature.

Pulsatile (PP) versus non-pulsatile (NP) flow during cardiopulmonary bypass (CPB) has been investigated by Dodonov and colleagues [4]. In their prospective randomized study, 52 patients were enrolled to evaluate the hemodynamic effects and endothelial reactivity of PP versus NP CPB. In the PP group, lower systemic and pulmonary vascular resistances were found, but a higher dosage of norepinephrine was required. Further studies are needed to better define the long-term effects of these two approaches during CPB. However, the data extrapolated by the Verona team are essential to growing the knowledge in this specific research field.

With the increased use of transfemoral approaches to perform minimally invasive cardiac surgery, Agostinelli and his co-authors [5] wanted to confirm the importance of the left ventricular apex as a safe and valid alternative to performing a variety of procedures such as valve repairs and replacements and thoracic aorta repairs.

A highly debated question was answered by Vendramin and his team [6] in the original work “Hemiarch Versus Arch Replacement in Acute Type A Aortic Dissection: Is the Occam’s Razor Principle Applicable?”. The authors analyzed the outcomes of 213 patients who underwent the repair of acute Type A aortic dissection (A-AAD), either by ascending aorta/hemiarch replacement or ascending aorta/total arch replacement. Survival at 5 and 10 years was worse in patients treated with a more conservative approach. In addition, the same group had a greater rate of reoperation events compared to the total arch replacement group. Therefore, the Udine (Italy) team demonstrated that an aggressive approach to A-AAD provides superior long-term results and should be considered the first-line approach in experienced centers.

To further highlight the variety of high-quality original papers published in this Special Issue, we would also like to mention the authors’ attention to investigating recent and new technologies. The left ventricular assist device Impella^®^ (Abiomed, Danvers, MA, USA) has been extensively implanted worldwide in acute cardiogenic shock patients. However, Zaiser and her team [7] published in this Special Issue one of the few original studies available in the literature that aims to analyze “the good and the bad” of this device. Out of the 281 included patients, 93% suffered from at least one adverse event. Moreover, half the patients suffered from acute kidney injury, making renal replacement therapy necessary in 35% of all patients. Therefore, due to the high rate of complications, this study highlights the importance of carefully selecting patients receiving micro axial LV support.

Lo Muzio and colleagues [8] demonstrated how artificial intelligence (AI) might play a vital role in the decision-making process during cardiac surgery operations. They focused on the intraoperative evaluation of the right ventricle by video-recording the beating hearts before and after pulmonary valve replacement in twelve Tetralogy of Fallot patients. The team demonstrated that AI could provide a real-time prognostic evaluation of RV functionality after pulmonary valve replacement; therefore, it may be an incredibly precious tool for surgeons.

In conclusion, we, the editors, strongly believe that this Special Issue may provide a tremendous amount of points of reflection for both younger and more experienced surgeons. Cardiac surgery is a fascinating field that is continually evolving, and research is necessary to feed the inquisitiveness that specific topics raise.

We would like to thank all the authors for their valuable contributions to this Special Issue. We hope to replicate this collaboration in the future to further explore the fascinating field of cardiovascular surgery.
